# Resolution-enhanced multi-core fiber imaging learned on a digital twin for cancer diagnosis

**DOI:** 10.1117/1.NPh.11.S1.S11505

**Published:** 2024-01-31

**Authors:** Tijue Wang, Jakob Dremel, Sven Richter, Witold Polanski, Ortrud Uckermann, Ilker Eyüpoglu, Jürgen W. Czarske, Robert Kuschmierz

**Affiliations:** a TU Dresden, Laboratory of Measurement and Sensor System Technique, Dresden, Germany; b TU Dresden, Competence Center BIOLAS, Dresden, Germany; c TU Dresden, Else Kröner Fresenius Center for Digital Health, Germany; d University Hospital Carl Gustav Carus, TU Dresden, Department of Neurosurgery, Dresden, Germany; e University Hospital Carl Gustav Carus, TU Dresden, Division of Medical Biology, Department of Psychiatry, Faculty of Medicine, Dresden, Germany; f TU Dresden, Excellence Cluster Physics of Life, Dresden, Germany; g TU Dresden, School of Science, Faculty of Physics, Dresden, Germany

**Keywords:** fiber bundle imaging, deep learning, tumor diagnosis

## Abstract

**Significance:**

Deep learning enables label-free all-optical biopsies and automated tissue classification. Endoscopic systems provide intraoperative diagnostics to deep tissue and speed up treatment without harmful tissue removal. However, conventional multi-core fiber (MCF) endoscopes suffer from low resolution and artifacts, which hinder tumor diagnostics.

**Aim:**

We introduce a method to enable unpixelated, high-resolution tumor imaging through a given MCF with a diameter of around 0.65 mm and arbitrary core arrangement and inhomogeneous transmissivity.

**Approach:**

Image reconstruction is based on deep learning and the digital twin concept of the single-reference-based simulation with inhomogeneous optical properties of MCF and transfer learning on a small experimental dataset of biological tissue. The reference provided physical information about the MCF during the training processes.

**Results:**

For the simulated data, hallucination caused by the MCF inhomogeneity was eliminated, and the averaged peak signal-to-noise ratio and structural similarity were increased from 11.2 dB and 0.20 to 23.4 dB and 0.74, respectively. By transfer learning, the metrics of independent test images experimentally acquired on glioblastoma tissue *ex vivo* can reach up to 31.6 dB and 0.97 with 14 fps computing speed.

**Conclusions:**

With the proposed approach, a single reference image was required in the pre-training stage and laborious acquisition of training data was bypassed. Validation on glioblastoma cryosections with transfer learning on only 50 image pairs showed the capability for high-resolution deep tissue retrieval and high clinical feasibility.

## 1 Introduction

Minimally invasive imaging is important to optogenetics^1^^–^^3^ and cancer diagnostics^4^^–^^6^ since it minimizes the damage to living tissues. Conventional brain cancer diagnosis requires surgical biopsy and resection, histological staining, and observation. The procedure is time-consuming, leading to treatment delay, and has no visual feedback during the surgery, which brings additional risk and complications.^7^^–^^9^ Label-free imaging techniques like autofluorescence^4^^–^^6^^,^^10^^–^^12^ and Raman spectroscopy^12^^–^^16^ enable locating target tissue *in situ* for *in vivo* tumor diagnosis,^17^^–^^19^ where high spatial resolution plays a critical role. Multi-core fibers (MCFs) are often used in endoscopy since they are flexible and ultra-thin (diameter<1  mm) and provide an efficient way to illuminate and detect in real-time,^20^^–^^24^ which allows minimal invasive access directly to deep tissue for intraoperative imaging. However, the fiber structure leads to honeycomb artifacts, which limit spatial resolution to the core-to-core spacing. Many approaches were proposed to enhance the resolution of fiber endoscopy, including physical methods,^25^^–^^28^ computational methods,^29^^–^^33^ and deep neural networks (DNNs).^34^^–^^40^ DNNs are advantageous because of their real-time capability, and no sophisticated optical systems are required.^41^

Convolutional neural networks (CNNs) greatly promote the development of image-based medical diagnosis in the last decade, e.g., surgical navigation^42^ and cancer recognition.^43^ Based on amounts of training data, CNNs can learn to extract, summarize, and reconstruct histomorphological features of tissue images using convolutional operations. In previous work, we proposed a near-video rate resolution enhancement method for MCF imaging, which enables all optical biopsies with minimal invasiveness.^34^ The learning-based approach inverts the image transmission properties for a given MCF-based endoscope. However, in reality, MCFs differ in core arrangement and transmissivity since glass fibers are not perfectly manufactured, leading to random and inhomogeneous optical properties. As a result, the DNN-based reconstruction requires experimental acquisition of an MCF-specific dataset, which is laborious and not easily transferable to clinics. Kim et al.^38^ proposed a reconstruction method for MCFs with random core arrangement, but the distortion resulting from inhomogeneous transmissivity and limited clinical data for training remains unsolved.

**Fig. 1:**
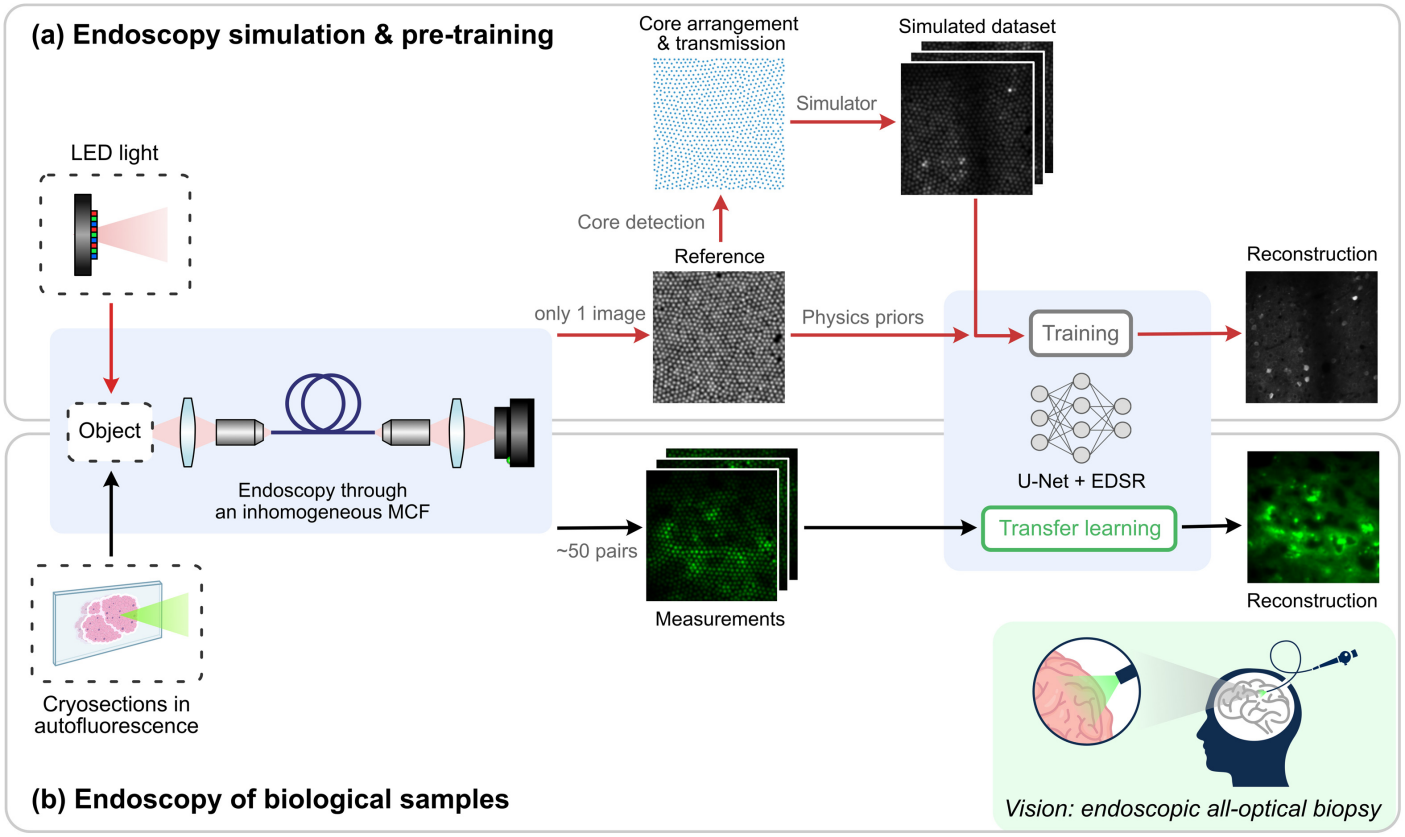
Digital twin concept for high-resolution image retrieval through a randomly selected MCF. (a) Single-reference-based endoscopy simulation and pre-training of U-Net + EDSR network. The MCF-specific reference provided physics priors of inhomogeneous optical properties of the MCF. (b) Endoscopy of biological samples in real-world contexts. Based on the pre-trained network, ∼50 autofluorescence image pairs of glioblastoma tissue through the same MCF as in (a) were collected for transfer learning.

Here, we present a streamlined process via a digital twin for MCF image retrieval with very few measurements of biological samples, as demonstrated in Fig. 1. In the pre-training, a single reference image of MCF was captured under incoherent widefield illumination, which offers physics priors of core arrangement and transmission for the data simulation. The reconstruction network was then pre-trained to remove honeycomb artifacts and enhance the image resolution. Subsequently, transfer learning was performed on 50 measured image pairs of brain tumor cryosections. Based on that, we demonstrate high-resolution MCF image retrieval on limited medical data, which is transferable in clinical practice and can significantly improve image-based tumor classification,^34^ for instance.

## 2 Methods

### 2.1 Reconstruction Network

A cascaded network consisting of a U-Net^44^ for depixelation and an enhanced deep super-resolution network (EDSR)^45^ for super-resolution was used. In previous work, this architecture was shown to enhance image resolution and benefit tumor classification. To make use of the physics priors of MCF, an extra input channel was added to the network transmissivity correction of MCF.

### 2.2 Simulated Dataset

The MCF dataset was simulated with the detected core arrangement and transmission of a randomly selected MCF (Fujikura FIGH-30-650S) based on a simulator.^46^ In total, 5,000 images from ImageNet^47^ were used for training, 100 for validation, and 400 for testing.

### 2.3 MCF Measurements

The reference image of the MCF used for the simulation was captured under incoherent widefield illumination, which provides core arrangement and transmission information for inhomogeneity correction and high-resolution retrieval. For validation, the autofluorescence images of cryosections of glioblastoma tissue prepared with a standard protocol^5^ were imaged through the same MCF as the reference. The samples were illuminated and imaged through the MCF using a 473 nm laser and camera CAM1 to emulate an endoscopic system, see Fig. 3(a). Autofluorescence was detected between 500 and 550 nm. High-resolution ground truth (GT) data was captured simultaneously with camera CAM2.

## 3 Results

**Fig. 2:**
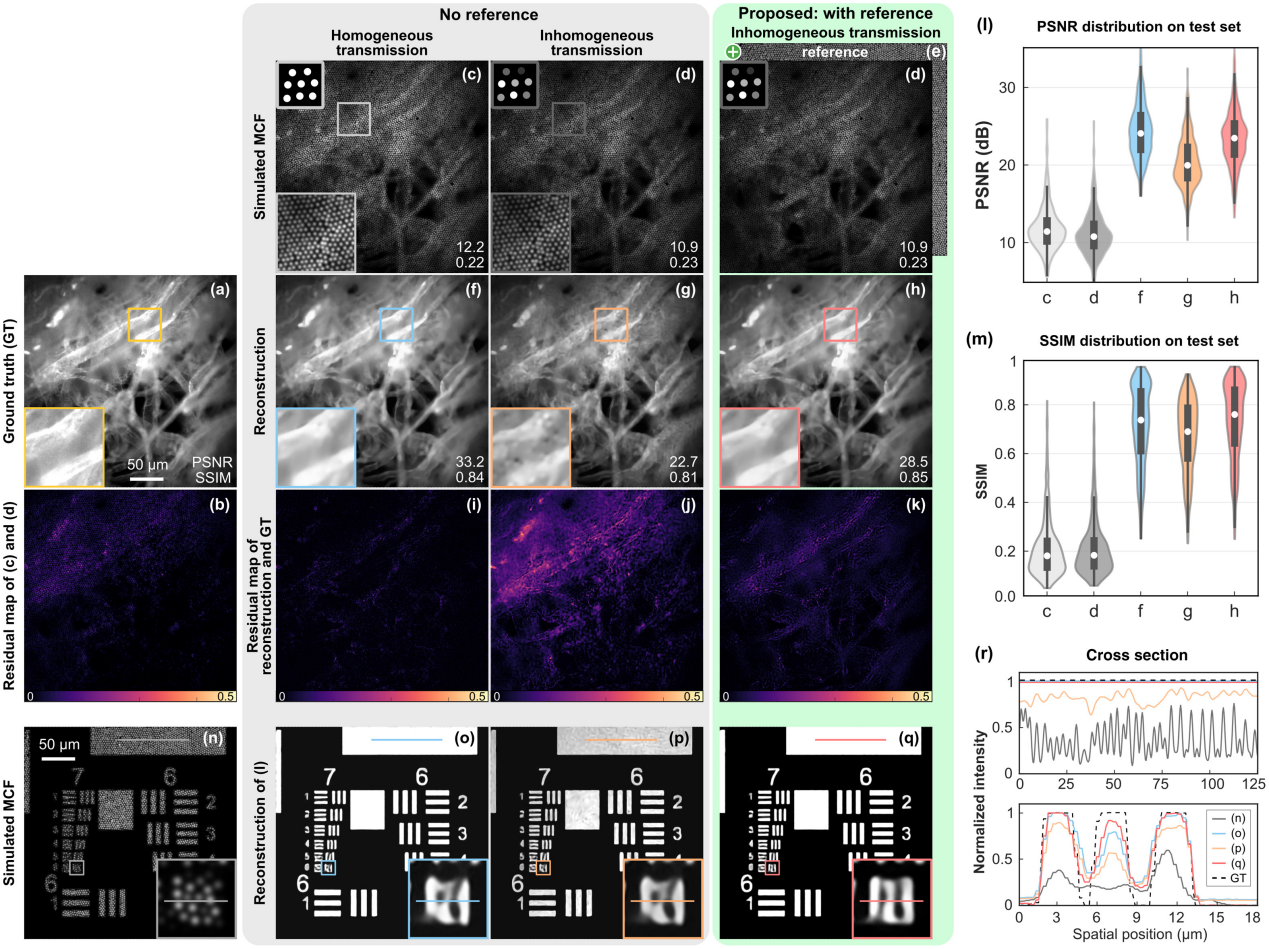
Retrieval of simulated MCF images by the pre-trained U-Net + EDSR network. (a) GT of a paper tissue instance. (b) Residual map of (c) and (d). (c) Simulated MCF image with homogeneous and (d) with inhomogeneous core intensity transmission. (e) Reference image containing core transmissivity as an additional input into the network. (f) and (g) Reconstructions of (c) and (d) by the no-reference-based network. (h) Reference-based reconstruction of (d). (i)–(k) Residual maps of (f)–(h) compared with GT. Although the visual difference of (c) and (d) is slight, (c) had a good reconstruction (f), while (d) resulted in image distortion in (g) by the same network. The distortion in (g)–(p), strongly depending on the inhomogeneous transmissivity, was eliminated by the reference-based approach with (e). (l) and (m) Quantitative image quality evaluation on the test sets in terms of PSNR and SSIM. The labels “c, d, f-h” in (m) correspond to the test sets of (c), (d), (f)–(h). (n)–(q) Simulated MCF image of a resolution test chart and the reconstructions using different approaches. (r) Cross section of the lines in (n)–(q).

The U-Net + EDSR model trained on the simulated MCF images of ImageNet was tested on two instances, paper tissue and resolution chart. Although these test image types had not been seen by the model during the training, the test results in Figs. 2(f) and 2(o) demonstrate good generalizability of the U-Net + EDSR. The reconstruction of a paper tissue image using the reference-based approach is shown in Fig. 2(h). For comparison, we present the results by the no-reference-based approach, namely the U-Net + EDSR with a single input, in Figs. 2(f) and 2(g). When an image through an inhomogeneous MCF was tested with the network trained on a homogeneous MCF dataset, distortion and hallucination appeared [see Fig. 2(g)]. The network did not learn how to correct the transmission inhomogeneity from the training data, consequently, the image quality of the reconstruction degraded significantly. In contrast, the reference-based approach learned priors containing MCF transmission information from the MCF-specific reference, where the average peak-to-noise ratio (PSNR) and structural similarity (SSIM) values of the test images are increased from 11.2 dB and 0.20 to 23.4 dB and 0.74, respectively, as shown in Figs. 2(l) and 2(m). The reconstruction of the resolution chart using different methods in Figs. 2(o)–2(q) demonstrates that the Group 7 Element 6 can be resolved by the reconstruction network. The cross sections in Fig. 2(r) show the imaging contrast.

**Fig. 3:**
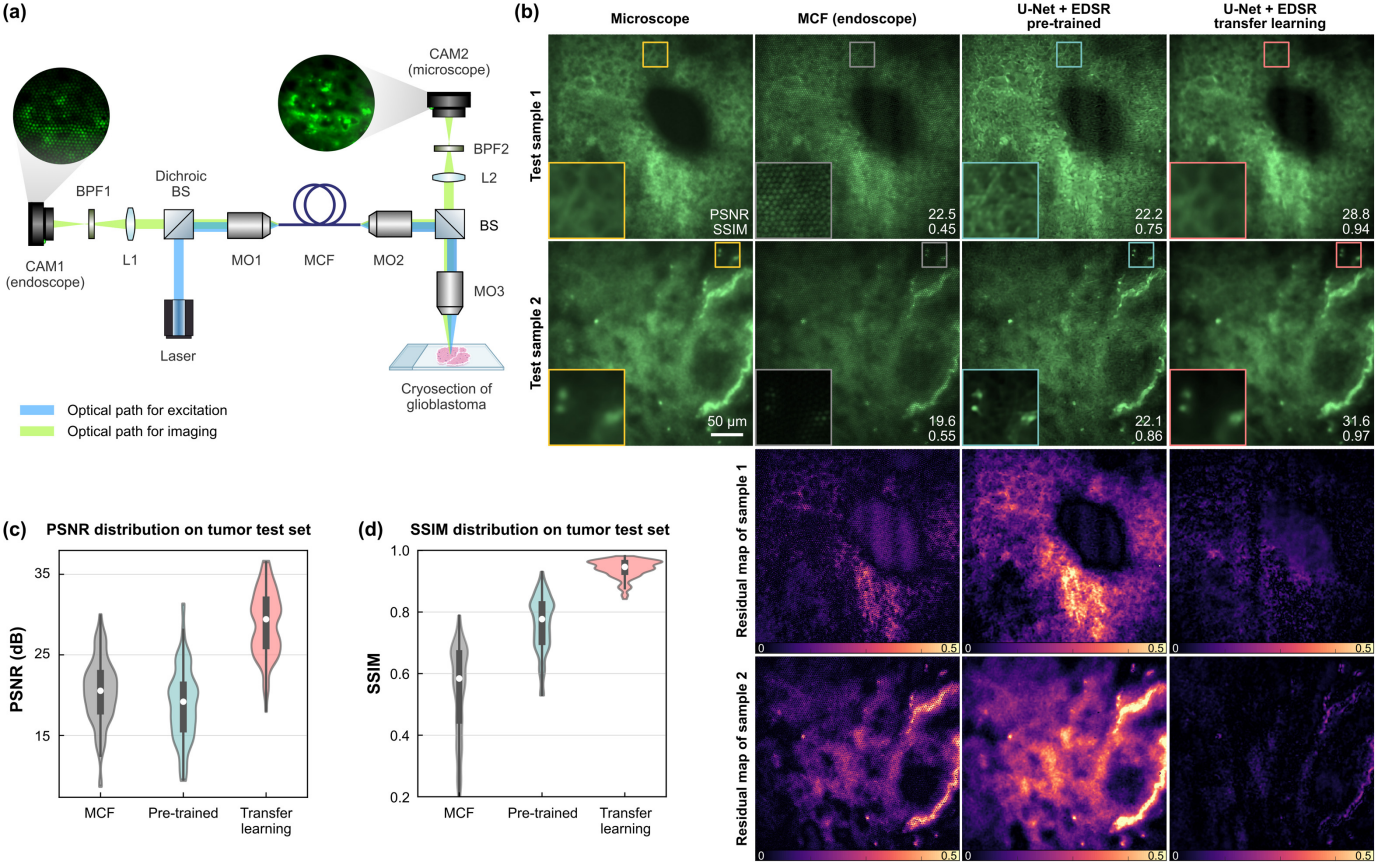
MCF image retrieval of glioblastoma cryosections with transfer learning. (a) Experimental setup for acquiring pairs of microscopic and endoscopic tumor images in autofluorescence with the same MCF as the reference image. CAM, camera; BPF, bandpass filter; L, lens; BS, beam splitter; MO, microscopic objective. (b) Qualitative comparison of image retrieval by the pre-trained network and transfer learning. Residual maps were obtained by comparing reconstruction results with microscopic images. The results solely using the pre-trained network were greatly distorted with artifacts. (c) and (d) Quantitative evaluation: PSNR and SSIM distribution evaluated on 94 measured MCF images of glioblastoma.

To further verify the retrieval of biological samples, the MCF was subsequently used for imaging cryosections of glioblastoma tissue. We captured the autofluorescence images of glioblastoma using the setup in Fig. 3(a), which combines a MCF endoscope and a widefield fluorescence microscope to capture both GT and measurement data, simultaneously. We used the MCF in this manner to improve the image reconstruction quality by transfer learning and validated the use of the proposed digital twin *ex vivo* for the application as an *in vivo* endoscope without additional optical elements. As demonstrated in Fig. 3(b), the results of the pre-trained network were distorted due to hallucination and artifacts remained. To eliminate the distortion, we used 50 pairs of captured microscopic and endoscopic glioblastoma images and applied transfer learning to the pre-trained network. Despite the limited data size, transfer learning was still able to further enhance image quality of glioblastoma tissue, and PSNR and SSIM values of the independent test images were increased up to 31.6 dB and 0.97, separately, with a near-video rate of 14 frames per second computing on a NVIDIA RTX A6000 GPU. The validation on biological samples shows that the reference-based approach enables retrieving high-resolution images even for a small experimental dataset which is easily obtainable in clinics.

## 4 Conclusions

DNNs enable high-resolution imaging through an MCF with micron resolution. This demands expensive data collection however, and the image reconstruction strongly depends on the optical properties of a given MCF. That means, experimental acquisition of thousands of MCF-specific image pairs is required for each single endoscope, which is not easily transferable to clinics. Here, a digital twin-based workflow is proposed to bypass costly acquisition of biological data by single-reference-based simulation of optical properties for an arbitrary MCF. Besides, the MCF-specific reference also provides physics priors of MCF inhomogeneity during training processes. The idea was validated on biological samples by transfer learning. Taking autofluorescence images of glioblastoma as an example, our approach can achieve precise retrieval on independent test images and improve PSNR and SSIM values up to 31.6 dB and 0.97, respectively, which required only 50 measured image pairs as training data (100 times less data than before). Our reference-based approach shows a high feasibility for clinical translation and is capable of image retrieval to improve image-based tumor classification during neurosurgeries.

## Data Availability

The data that support the findings of this article are not publicly available due to ethical concerns. A part of them is available from the author upon reasonable request.
